# Isolated Pyridoxine Deficiency Presenting as Peripheral Neuropathy Post-chemotherapy

**DOI:** 10.7759/cureus.26725

**Published:** 2022-07-10

**Authors:** Aanchal Sawhney, Sachi Singhal, Rahul Patel

**Affiliations:** 1 Internal Medicine, Crozer-Chester Medical Center, Upland, USA

**Keywords:** chemotherapy-induced polyneuropathy, pyridoxal kinase, carboplatin-etoposide, peripheral neuropathy, pyridoxine deficiency

## Abstract

Pyridoxine deficiency is a rare but identifiable cause of sideroblastic anemia, depression, and peripheral neuropathy. Platinum-based chemotherapeutic drugs display structural similarity to pyridoxine, which interferes with the absorption and hence the efficacy of the drug. If left untreated, it can lead to irreversible axonal loss and permanent deficits, leading to falls. Our case is a highly unusual scenario of isolated pyridoxine deficiency presenting as peripheral neuropathy and depression as a delayed side effect of chemotherapeutic drugs.

## Introduction

As reported by Barrell et al., peripheral neuropathies encompass a broad range of disorders affecting the peripheral nervous system including distal sensory neuropathy, mononeuritis multiplex, polyradiculopathies, and neuropathy causing non-length-dependent pan-modal sensory loss or weakness [1]. The most common pattern is distal sensory polyneuropathy (DSP), associated with length-dependent peripheral nerve injury resulting in distal predominant sensory loss, pain, and severe weakness, resulting in gait instability, fall risk, and, in some instances, foot ulceration and amputations [1]. The common causes of neuropathy include diabetes-associated neuropathy, and vitamin B12 and folate deficiency. Isolated pyridoxine deficiency presenting as peripheral neuropathy has been sparsely reported in case reports.

Pyridoxine deficiency is often associated with concomitant deficiencies of other water-soluble vitamins such as vitamin B12 and folate [2]. Isolated pyridoxine deficiency is very rare in the United States. It is associated with conditions of increased metabolism such as renal impairment on hemodialysis or peritoneal dialysis, autoimmune conditions such as rheumatoid arthritis, and states of decreased consumption/absorption (pregnancy, protein-energy malnutrition, chronic alcohol dependence, celiac disease, or post-bariatric surgery) [3].

## Case presentation

A 67-year-old male presented to our clinic with the chief complaint of numbness in his legs. He first noticed this when he was getting a hot stone massage and could not appreciate the heat over his legs up to his knees. However, he could feel the hot stones over his thighs and everywhere else on his body. He also reported associated tingling in his toes up to his knees. He could ambulate without difficulty and denied any recent trauma to his legs. He lived at home and was currently not working. He did not recall any exposure to heavy metals or industrial toxins in the past. He was a current smoker and smoked half a pack a day and denied any other substance use. He had never been tested for HIV and was sexually active with two partners with intermittent barrier contraception use. His past medical history included non-small cell lung cancer (NSCLC) in remission post-chemoradiation, chronic prostatitis, prediabetes, anxiety, osteoarthritis, and depression. The NSCLC was treated three years ago, with a six-month course of carboplatin-etoposide (every four weeks), and has been in remission for one year now. His physical examination revealed impaired vibratory sensations bilaterally from his feet up to both knees. He also had an impaired sense of temperature spanning the same region. His gait was unremarkable, and the rest of his neurological, cardiac, pulmonary, and gastrointestinal examinations was benign.

A broad workup was initiated, and his cell counts, blood chemistries, liver function tests, HbA1c, and sexually transmitted disease panel including syphilis, gonorrhea, chlamydia, HIV, and hepatitis panels came back within normal limits. Given a history of chemotherapy and an unremarkable initial workup, he was also evaluated for thiamine, pyridoxine, and cobalamin deficiency. As presented in Table 1, his thiamine and cobalamin levels were normal, and pyridoxine resulted at an abnormally low level of 1.6 µg/L and was the only plausible explanation of the patient’s peripheral neuropathy, concomitantly explaining the diagnosis of depression. The patient was placed on oral repletion with pyridoxine 100 mg once daily with improvement noted within three months of initiation.

**Table 1:** Blood workup of peripheral neuropathy

Test	Result	Reference range
Pyridoxine levels	1.6 µg/L	5-50 µg/L
Thiamine levels	6.1 µg/dL	2.5-7.5 µg/dL
Cobalamin levels	350 pg/mL	160-950 pg/mL
Hemoglobin A1c	5.6%	<5.7%
HIV Ab	Negative	Negative
HIV p24 antigen	Negative	Negative
HBs Ag	Negative	Negative
Hep B Core Ab	Negative	Negative
HCV titer	<0.1	0.0-0.9
Chlamydia NAAT	Non-reactive	Non-reactive
Neisseria NAAT	Non-reactive	Non-reactive
RPR	Non-reactive	Non-reactive

Utilizing a stepwise approach to evaluate the cause of our patient’s subacute/insidious acquired, symmetrical, distal, focal, sensory neuropathy, a diagnosis of isolated pyridoxine deficiency was clinched and managed.

## Discussion

Neuropathy, if left unattended, can lead to irreversible axonal loss and permanent deficits. Hence, identifying the correct etiology is imperative for the prevention of further damage, as well as potential reversal and management of the neuropathy. Unlike plants and bacteria, mammalian cells cannot synthesize pyridoxine, and dietary intake with meat and vegetables along with production by the gut microflora is the source of pyridoxine for humans [4]. The dietary allowance for pyridoxine is 1-2 mg for adults, and the absorption is unsaturated [4]. Pyridoxine, similar to cyanocobalamin and folate, is required in the metabolism of methionine to cysteine and hence necessary for neuronal survival [4]. Pyridoxine deficiency is known to impair transcellular signaling between neurons and usually presents itself as a culmination of various nonspecific symptoms sometimes difficult to put together [3]. Presenting features include depression, anemia, muscular convulsions, hyperirritability, and peripheral neuropathy [3,5].

The causes of neuropathy can be classified into two broad categories, hereditary and acquired, the former including Charcot-Marie-Tooth (CMT) disease, neuropathy hereditary transthyretin amyloidosis, porphyria, Fabry disease, Refsum disease, and metachromatic leukodystrophy. Acquired causes include drugs, toxins, immune-mediated, infectious, cancer-related, compressive, and metabolic conditions [6]. Some drug-related causes of pyridoxine deficiency include isoniazid, penicillamine, levodopa, and chemotherapeutic drugs, which interfere with its metabolism.

The presence of peripheral neuropathy secondary to pyridoxine deficiency in the absence of sideroblastic anemia, similar to our patient, is a rare but known presentation of pyridoxine deficiency, which is reported in the literature but easily missed in patient evaluation [3]. Previously reported in the literature is a case report with isolated pyridoxine deficiency in a diabetic patient, presenting solely as muscle spasms [5].

Carboplatin is a platinum-based chemotherapeutic drug used for the treatment of solid tumors, which bind to DNA nucleobases, leading to replication and transcription suppression, apoptosis, or necrosis in tumor cells [7]. The commonly seen adverse effects include myelosuppression, renal insufficiency, and peripheral neuropathy seen in 4%-6% of patients posttreatment [8,9]. Spectroscopic studies have revealed complex formation between pyridoxine and carboplatin due to structural similarity to adenine and guanine, leading to a decrease in the maximum absorbance of pyridoxine relative to its baseline [7]. In an experimental study on NSCLC, it was found that pyridoxine and pyridoxal kinase (PDXK) exacerbates, in a PDXK-dependent manner, cisplatin cytotoxicity in vitro and in vivo. The expression of PDXK levels functions as a prognostic biomarker for risk stratification among patients with NSCLC. Low levels have been associated with poor outcomes [10].

Pyridoxine in high doses interferes with the efficacy of chemotherapeutic drugs and can also precipitate neuropathy [11]. Adequate management of pyridoxine deficiency in at-risk patients includes a daily dose of 6 mg of pyridoxine. Daily dosing greater than 50 mg/day if used for longer than six months has been proven harmful [4,12].

## Conclusions

This case emphasizes identifying pyridoxine deficiency as a rare but known cause of peripheral neuropathy in the population at risk and monitored dosing of the vitamin due to its narrow therapeutic index. Prophylactic supplementation with vitamin B12 in patients undergoing chemotherapy is considered an economical and safe option for the prevention of cisplatin-induced peripheral neuropathy (CIPN) development. However, it is a tricky situation with pyridoxine supplementation as, in high doses, it not only interferes with the efficacy of chemotherapeutic drugs but can also precipitate neuropathy; therefore, pyridoxine testing is necessary before treating prophylactically. High suspicion of chemotherapy-induced peripheral neuropathy secondary to pyridoxine deficiency should be present even in delayed presentation of neuropathy.
